# Site I Inactivation Impacts Calmodulin Calcium Binding and Activation of *Bordetella* *pertussis* Adenylate Cyclase Toxin

**DOI:** 10.3390/toxins9120389

**Published:** 2017-11-30

**Authors:** Christian W. Johns, Natosha L. Finley

**Affiliations:** 1 Cell, Molecular, and Structural Biology Program, Miami University, Oxford, OH 45056, USA; johnscw@miamioh.edu; 2 Department of Microbiology, Miami University, Oxford, OH 45056, USA

**Keywords:** calcium, calmodulin, CyaA toxin, NMR

## Abstract

Site I inactivation of calmodulin (CaM) was used to examine the importance of aspartic acid 22 at position 3 in CaM calcium binding, protein folding, and activation of the *Bordetella pertussis* adenylate cyclase toxin domain (CyaA-ACD). NMR calcium titration experiments showed that site I in the CaM mutant (D22A) remained largely unperturbed, while sites II, III, and IV exhibited calcium-induced conformational changes similar to wild-type CaM (CaMWt). Circular dichroism analyses revealed that D22A had comparable *α*-helical content to CaMWt, and only modest differences in *α*-helical composition were detected between CaMWt-CyaA-ACD and D22A-CyaA-ACD complexes. However, the thermal stability of the D22A-CyaA-ACD complex was reduced, as compared to the CaMWt-CyaA-ACD complex. Moreover, CaM-dependent activity of CyaA-ACD decreased 87% in the presence of D22A. Taken together, our findings provide evidence that D22A engages CyaA-ACD, likely through *C*-terminal mediated binding, and that site I inactivation exerts functional effects through the modification of stabilizing interactions that occur between *N*-terminal CaM and CyaA-ACD.

## 1. Introduction

The etiological agent of whooping cough, *Bordetella pertussis* (Bp), utilizes the adenylate cyclase toxin (CyaA) to impair the host immune response and facilitate the establishment of respiratory infections [1,2,3,4]. This reemerging pathogen is a global public health concern, and has prompted renewed interest in finding novel therapeutic approaches to combat whooping cough, also known as pertussis. Because of its importance as a Bp virulence factor, CyaA has been identified as a potential pharmaceutical target for the development of selective inhibitory compounds [5,6,7,8]. Moreover, increasing concern about antibiotic resistance in Bp [9] emphasizes the need to combat drug resistance in microorganisms by discovering new treatment strategies that might involve targeting bacterial virulence factors such as CyaA at the molecular level. 

CyaA consists of the *C*-terminal hemolysin domain (141 kDa) and the *N*-terminal adenylate cyclase domain (43 kDa) (CyaA-ACD) [1,10,11]. During host infection, CyaA is activated by the eukaryotic calcium (Ca^2+^)-sensing protein calmodulin (CaM), which leads to the intracellular elevation of cyclic adenosine monophosphate (cAMP) and subsequent suppression of the immune response. Bacteria lacking CyaA are more susceptible to destruction by the immune system, suggesting that the presence of functional CyaA is critical to pathogenesis [3,12], but the molecular details of CyaA activation remain unclear.

CyaA-ACD shares structural and functional homology with bacterial adenylate cyclase toxins from *Bacillus anthracis* (edema factor-EF) [13] and *Pseudomonas aeruginosa* (ExoY) [14], but relatively little amino acid conservative exists between these proteins. Cytoskeletal actin is the host cell activator of ExoY [15], whereas EF and CyaA are stimulated by site-specific contacts mediated through differential interactions with the *N*-terminal and *C*-terminal domains of CaM [10,16]. The helical domain of EF engages *N*-terminal CaM, while the *C*-terminal domain of CaM is important in providing a hydrophobic binding interface with helix H of EF [13]. No high-resolution structure of CyaA, either free or bound to intact CaM, is available; but X-ray crystal structures of *C*-terminal CaM bound to CyaA-ACD confirm that helix H of CyaA-ACD also plays a significant role in *C*-terminal CaM association [17]. It is known that maximal enzymatic activity of CyaA is stimulated through interaction with both the *N*-terminal and *C*-terminal lobes of CaM [18]. Intermolecular association has been reported between *N*-terminal CaM and CyaA-ACD [19], and mutation in CyaA-ACD disrupts this low-affinity protein-protein binding interface [20,21]; however, it remains to be determined how activation of CyaA-ACD is achieved through association with both domains of CaM.

Human CaM is an acidic protein composed of 17 aspartic acids and 21 glutamic acids. The *N*-terminal and *C*-terminal domains of CaM have hydrophobic clefts important in target binding that are connected by a flexible linker. Each domain is composed of two highly conserved Ca^2+^/Mg^2+^-binding sites consisting of consecutive amino acid residues that provide oxygen ligands for metal coordination. In the *N*-terminal domain, positions 1, 3, 5, 7, 9, and 12, corresponding to residues D20, D22, D24, T26, T28, and E31, respectively, in site I, and residues D56, D58, N60, T62, D64, and E67, respectively, in site II, ligate divalent metals directly [22]. In the *C*-terminal domain, sites III and IV are higher-affinity metal binding sites that utilize similar ligands as sites I and II, which contribute to target recognition through metal-induced conformational changes in CaM [23,24]. The metal occupancy of CaM can be monitored by NMR using the chemical shift values at position 8 (I27, I63, I100, and V136 respectively) in sites I-IV [25], which can be a useful tool in studying the Ca^2+^-induced conformational changes critical in target binding.

While *C*-terminal CaM association involving bound Ca^2+^ accounts for the high-affinity interaction with CyaA-ACD [26], studies have shown that inter-domain communication exists in CaM in the presence of CyaA-ACD and ligated metals [21], revealing the importance of understanding how intact CaM activates CyaA-ACD. Previously, it was reported that several residues spanning from amino acid 22 to 38 in CaM, including site I and the linker between helices 2 and 3, are involved in direct protein-protein contacts with CyaA-ACD [20,21]; but it is not understood how intermolecular binding and metal ligation in this region of CaM impact its ability to fully activate CyaA-ACD. The potential involvement of site I at position 3 in CyaA-ACD binding was particularly interesting, as one carboxylate oxygen of D22 directly coordinates metal, while the remaining one is solvent exposed in free CaM [22]. Based on these observations, we speculated that D22 in CaM may serve as one potential contact point for CyaA-ACD engagement that is also sensitive to Ca^2+^-induced changes in CaM.

The purpose of this study was to utilize nuclear magnetic resonance (NMR), circular dichroism (CD), and functional assays to examine how mutation at D22 in site I (aspartic acid to alanine (D22A)) of CaM impacts its Ca^2+^-binding properties, protein folding characteristic, and its activation potential of CyaA-ACD. With the D22A mutation, we predicted that replacement of the aspartic acid with an alanine would disrupt metal binding at site I and possibly reduce toxin activation through modulation of site-specific intermolecular association between CaM and CyaA-ACD.

## 2. Results

### 2.1. Metal Binding to D22A

**Figure 1:**
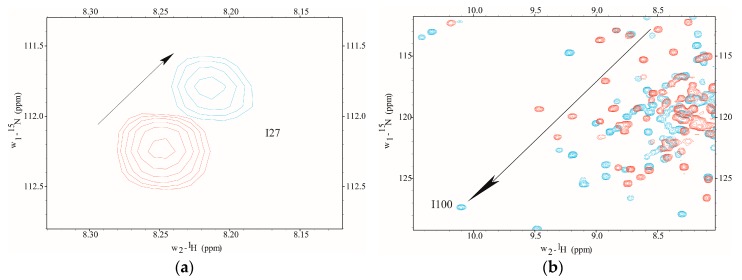
2D ^1^H-^15^N correlation spectra collected during NMR Ca^2+^ titration (Apo D22A (Red) and Ca^2+^-loaded D22A (Cyan)). Position 8 (residue I27) in metal binding site I does not exhibit significant Ca^2+^-dependent conformational changes (**a**). At position 8 in site III (residue I100), D22A has Ca^2+^-induced chemical shift changes that are indicative of metal coordination and are similar in magnitude to those observed for CaMWt (**b**). Arrows show direction of chemical shift change during Ca^2+^-titration.

The Ca^2+^-binding was monitored using 2D NMR to determine the amino proton-nitrogen chemical shifts for each amino acid residue mapping to metal binding sites I–IV in D22A. NMR chemical shift assignments were achieved by direct spectral comparison to previously reported values [20,21]. In the current study, we found that site-specific metal binding to D22A was evidenced by the direction and magnitude of amide proton-nitrogen chemical shift values observed in the presence of Ca^2+^-saturation (Figure 1). However, the addition of Ca^2+^ to D22A did not induce chemical shift perturbations at position 8 (I27), the metal occupancy reporter residue in CaM, an observation which is indicative of site I inactivation (Figure 1a). In contrast, Ca^2+^-induced downfield chemical shift changes were detected at position 8 (I100) in site III of D22A, which suggests that Ca^2+^-ligation in the *C*-terminal domain occurs in a manner comparable to CaMWt (Figure 1b).

**Figure 2:**
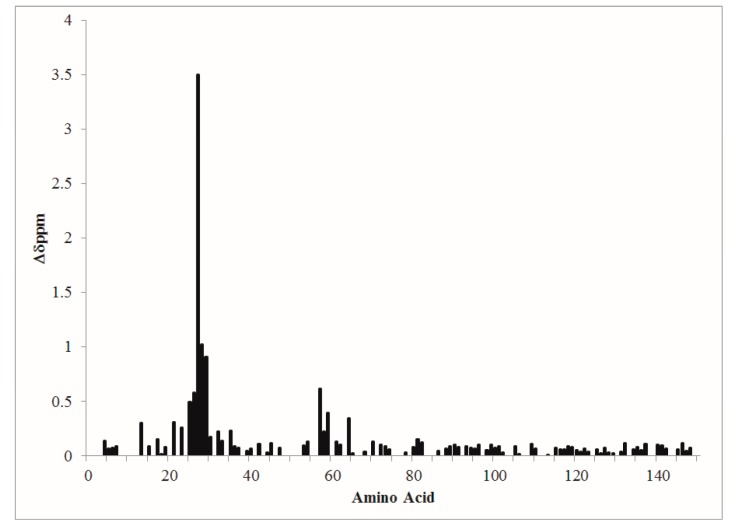
Composite amide-proton nitrogen chemical shift changes are plotted versus CaM amino acid sequence.

Composite amide proton–nitrogen chemical shift mapping revealed that the global conformations of D22A and CaMWt were very similar and that structural perturbation of D22A was localized primarily to site I (Figure 2).

Most notably, position 8 in site I of D22A did not exhibit values of chemical shift consistent with Ca^2+^-ligation. Minor perturbations were observed in site II, while the conformation at sites III and IV was largely the same between D22A and CaMWt in the presence of Ca^2+^.

### 2.2. Secondary Structure and Thermal Stability

**Figure 3:**
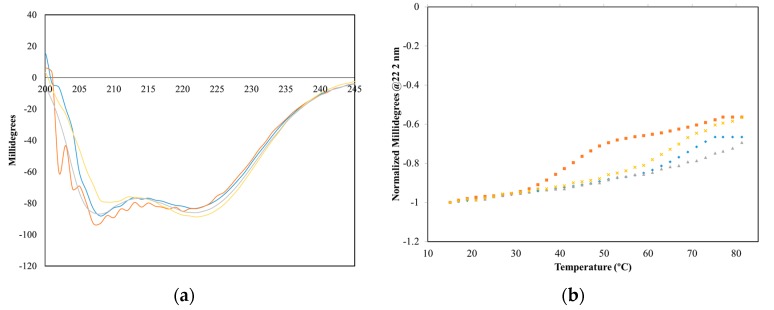
Far-UV CD spectra collected at 25 °C: Far-UV scan of (**a**) D22A (gold), D22A/CyaA-ACD (orange), CaMWt (gray), CaMWt/CyaA-ACD (blue); (**b**) Thermal denaturation of proteins monitored at 222 nm.

The global secondary structure of D22A free and bound CyaA-ACD was monitored using CD. Both D22A and CaMWt had high global *α*-helical content, as evidenced by the ratio of *θ*_222_/*θ*_208_ (Table S1), suggesting that the absence of Ca^2+^-binding at site I had minor conformational consequences in the *N*-terminal domain (Figure 3a; Table S1). Spectra recorded for CaMWt/CyaA-ACD showed distinct secondary structure composition and *θ*_222_/*θ*_208_ ratio as compared to free samples (Table S1), which is consistent with conformational change occurring upon formation of the protein-protein complex. The observation of distinct CD spectra in the D22A/CyaA-ACD was indicative of a conformational change occurring in the complex sample. Similarities between CD spectra of D22A/CyaA-ACD and CaMWt/CyaA-ACD were observed, which supported the notion that D22A also engages CyaA-ACD. The measurement of a lower *θ*_222_/*θ*_208_ ratio for D22A/CyaA-ACD was interpreted to mean that this complex likely has less *α*-helical content than CaMWt/CyaA-ACD.

The thermal stability of D22A was monitored in the presence and absence of CyaA-ACD. It was determined that mutation at position 3 in site I induces a slightly different thermal transition pattern as compared to CaMWt. In the presence of CyaA-ACD, greater thermal instability was observed, as evidenced by a lower transition temperature in the D22A/CyaA-ACD complex as compared to CaMWt/CyaA-ACD (Figure 3b). The loss of Ca^2+^-binding at site I in D22A affected the unfolding of the complex, emphasizing the importance of this region in stabilizing CyaA-ACD interaction.

### 2.3. CaM-Dependent CyaA Activation

**Figure 4:**
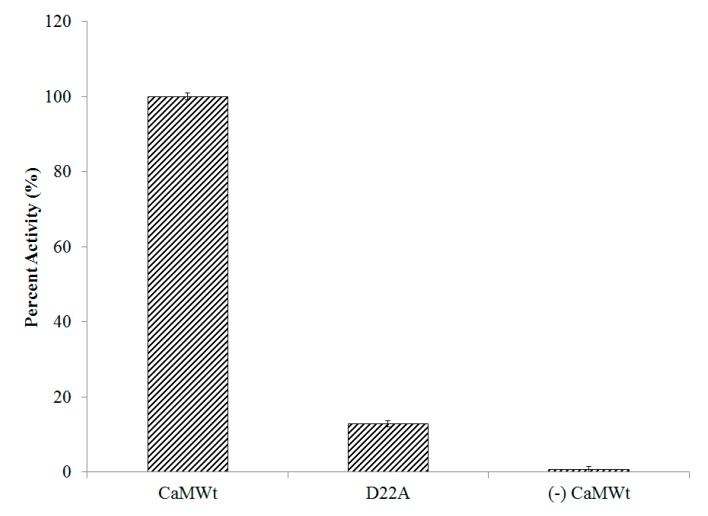
Site I inactivation in D22A reduces CyaA-ACD activity. Complexes consisting of CaMWt/CyaA-ACD were considered to have 100% enzymatic activity, whereas stimulation in the absence of CaMWt activator was a measure of basal level function. The activity reported for each sample was an average and the standard error calculated for three independent measurements each collected in triplicate.

The functional consequence of site I inactivation was investigated by measuring the enzymatic activity of CyaA-ACD in the presence of D22A (Figure 4). The basal level of CyaA-ACD activation in the absence of the host activator was measured, and was determined to be marginal. In the presence of CaMWt, full CyaA-ACD activation was considered to 100%. We observed a substantial reduction in activity of 87% in complexes consisting of D22A/CyaA-ACD, suggesting that defunct Ca^2+^-binding site I impacts activation of CyaA-ACD.

## 3. Discussion

The functional importance of intact CaMWt in the maximal activation of CyaA-ACD is known [18]. Despite many years of intensive investigation into the structural mechanism regulating CaM-dependent activation of CyaA-ACD, there is no available high-resolution structure of CyaA-ACD, either free or bound to intact CaM. In a partial crystal structure, CyaA-ACD is bound to *C*-terminal CaM, which offers mechanistic insight into the function, but the *N*-terminal CaM is absent, so its role in activation is still incompletely understood [17]. Difficulty in obtaining a high-resolution structure is likely a consequence of inherent flexibility in the CaM/CyaA-ACD complex, which precludes structure determination. Moreover, CyaA-ACD has been recalcitrant to solution studies due to its tendency to aggregate in the absence of CaMWt. Numerous biochemical, computational, and biophysical studies have been performed that have provided critical information regarding the structure and function of CyaA in the presence and absence of CaM [19,27,28,29]. Previously, it has been shown that direct protein-protein association occurs between CyaA-ACD and amino acid residues located near site I of CaM. Furthermore, mutations in *N*-terminal CaM or the β-hairpin of CyaA-ACD modulate the global conformation of the CaM/CyaA-ACD [20,21]. 

In this study, we utilized NMR, CD, and biochemical assays to examine the influence of position 3 in site I of CaM on CyaA-ACD activation. The region near position 3 in CaM was identified as a site of intermolecular association with CyaA-ACD [20]. At this position, D22 contributes a carboxylate ligand for Ca^2+^-coordination in CaM [22], but its significance in CyaA-ACD interaction is unclear. Using 2D NMR and composite amide proton-nitrogen chemical shift mapping, we showed that site I in D22A was defunct while site III and IV in the *C*-terminal domain of CaM exhibited chemical shift differences consistent with Ca^2+^-coordination. Moreover, the chemical shift differences between CaMWt and D22A were localized primarily to site I in the *N*-terminal domain, suggesting that the *C*-terminal domains are structurally similar. Taken together, our findings supported that D22A does not have undesired conformational changes, and thus is a suitable mutant for studying *N*-terminal effects of CaM association on CyaA-ACD activation.

Even with the dysfunctional Ca^2+^-binding site I, D22A had high relative *α*-helical content similar to CaMWt. This observation is consistent with reports of only minor changes in secondary structure occurring upon Ca^2+^-saturation of CaMWt [30]. In general, spectra for both complexes are consistent with those published for CaM/CyaA-ACD complexes [28]. Far-UV CD analyses indicated that D22A forms a complex with CyaA-ACD that is conformationally distinct from free CaM, but the mutant complex displays some observable differences in *α*-helical content as compared to CaMWt/CyaA-ACD. The ratio of *θ*_222_/*θ*_208_ has been utilized to characterize changes in *α*-helical content of CaM in the presence and absence of effector proteins [30]. We used this approach to show that D22A/CyaA-ACD exhibited a lowered *θ*_222_/*θ*_208_ ratio and less thermal stability than CaMWt/CyaA-ACD; in combination these findings suggest that disruption of site I in D22A destabilizes complex formation. In similar studies, it was found that alteration of residues at the protein interface between *N*-terminal CaM and CyaA-ACD disrupts the complex in a site-specific manner [20,21]. It is also possible that disruption of the *N*-terminal binding interface with CyaA-ACD modulates any conformational stability induced in CyaA-ACD upon CaM association.

Finally, we tested the impact of site I inactivation on CyaA-ACD stimulation. We found that association with D22A reduced CyaA-ACD activation, which is consistent with this region of CaM playing a direct role in enzymatic stimulation by stabilizing interactions between the activator and the toxin. To our knowledge, this is the first report that site I inactivation at this position in the metal binding loop of CaM diminishes CyaA-ACD activity. Previously, it was shown by NMR that the *N*-terminal and *C*-terminal domains of CaM are in more “open” conformations in the presence of CyaA-ACD [20]. This is in direct contrast to the complex formed between EF and CaM, whereby *N*-terminal CaM remains in a more “closed” state as a consequence of association with the helical domain of EF [31]. The importance of metal binding sites in *C*-terminal CaM has also been reported [32], which supports the concept that adenylate cyclase toxins might have evolved to have specialized features that would permit them modulate the Ca^2+^-sensing properties of their host cell activator CaM. Since both EF and CyaA-ACD have been shown to interact with *N*-terminal CaM near metal-binding sites, and it is known that this interaction can affect the degree of hydrophobic exposure in the lobes of CaM, it is interesting to speculate on the role of conformational transitions in the bacterial adenylate cyclase toxin activation. In other EF-hand proteins, target binding is known to invoke a disorder to order transition in effector proteins [33] and to modulate domain conformational plasticity, both of which are recognized as means by which to fine-tune Ca^2+^ sensitivity in biological systems [34,35].

In summary, our findings demonstrate that interaction with site I of CaM is a mechanism by which the potency of CyaA-ACD activation can be modified. The *C*-terminal domain of CaM has high affinity for CyaA-ACD, while the *N*-terminal domain engages CyaA-ACD through low-affinity binding. It is possible that these low-affinity interactions permit transient association between CaM and CyaA-ACD, allowing for more rapid response to fluctuations in the intracellular Ca^2+^ concentrations. We propose that bacterial adenylate cyclase toxins exploit the modulation of Ca^2+^-sensing activity in its activator protein CaM as a means by which to regulate its stimulation within the host cell environment. Future work in this field should include an examination of how metal-sensing capabilities and conformational plasticity function in CaM-dependent activation of CyaA, as this information is likely relevant to understanding how other bacterial adenylate cyclase toxins work. Expanded structural knowledge of CaM-dependent CyaA-ACD activation is necessary to design selective inhibitors for novel therapeutic approaches, which may be important in the study of other CaM-dependent enzymes as well.

## 4. Materials and Methods

### 4.1. Sample Preparation, Sodium Dodecyl Sulfate Polyacrylamide Gel Electrophoreses (SDS-PAGE), and NMR Analyses of Recombinant Protein

Recombinant CyaA-ACD and CaM were overproduced and purified as previously described [20,21]. Site-directed mutagenesis of D22A was performed according to the manufacturer’s protocol using the Quikchange^TM^ mutagenesis kit. Recombinant stable isotope labeled and unlabeled D22A were expressed and purified as previously described [20,21]. UV absorption and Bradford assay was used to quantify purified CaMWt, D22A, and CyaA-ACD proteins. 

Prior to NMR studies, samples of [^15^N] ApoD22A (0.5–0.7 mM in concentration) were stored in plastic conical tubes in the following buffer: 250 mM NaCl, 1 mM EGTA, 20 mM Hepes-NaOH pH 7.3, 1 mM PMSF, and 10% D_2_O (NMR buffer). NMR experiments were carried out using a Bruker Avance III 600 MHz spectrometer equipped with a conventional 5 mm probe. The NMR chemical shift assignments were transferred from previous experimental data [20,21]. Transverse Relaxation Optimized (^1^H-^15^N TROSY-HSQC) 2D spectra were collected for [^15^N]D22A at 298 K in the presence and absence of Ca^2+^-saturation (final concentration 12 mM). To confirm the chemical shift assignments for [^15^N]D22A, ^15^N edited NOESY-HSQC multidimensional NMR experiments were collected using 70 ms mixing time. NMR data were processed using NMRPipe [36], and analyzed using Sparky [37] and TopSpin 3.2. Amide proton-nitrogen chemical shift perturbations were calculated as previously described [20,21].

### 4.2. CD Analyses

CD spectra of were collected using a spectropolarimeter (Aviv model 435; Aviv Biomedical, Lakewood, NJ, USA). Data were recorded between 260 nm and 185 nm using a quartz cuvette with a 0.1 cm path length equipped with temperature control. Spectra were an average of 10 scans and were collected at 1 nm intervals using scan speeds of 50 nm/min. During thermal denaturation experiments, the temperature was increased from 25 °C to 85 °C, with ellipticity measurements being recorded at 5.0 °C intervals for all CD analyses, protein samples were suspended in 10 mM potassium phosphate (pH 7.5) and 0.25 mM CaCl_2_ at 0.25 mg/mL. Prior to data analysis, background corrections were performed by subtracting baseline buffer spectra from the experimental data. The thermal denaturation of the proteins was monitored at 222 nm using a rate of 2.00 °C/mine and an incubation time of 100 s from 15 °C to 95 °C. For each sample, three measurements were performed, and the average normalized signals are presented. 

### 4.3. Activity Assays

The PiColorLock Gold Phosphate Detection System (Innova Biosciences, Cambridge, UK) was used to measure the enzymatic activity of CyaA-ACD in the presence and absence of CaMWt and D22A according to the manufacturer’s suggested protocol. The assay was carried in microplates using CyaA-ACD suspended in at a final concentration of 0.2 nM in 20 mM Hepes-NaOH pH 7.3, 15 mM MgCl_2_, 0.2 mM CaCl_2_, and 0.01% Tween (AC buffer) in the presence of 4 units/mL of pyrophosphatase. To the reaction mixture, CaM was added, to a final concentration of 2.0 µM; the plates were incubated for 10 min at 30 °C, and ATP was added to a final concentration of 2 mM. Incubation at 30 °C was continued for 15 min. From each reaction mixture, 10 µL samples were transferred to a clean microplate well with 40 µL of fresh AC buffer and 12.5 µL of Gold Mix. Following incubation at room temperature for 5 min, 5 µL of stabilizer reagent was added and the optical density was recorded at 595 nm after 30 min. The experimental assay values were converted to moles of pyrophosphate produced by using a standard curve of known phosphate concentrations. Each protein sample was assayed with three independent measurements, each performed in triplicated. The mean enzymatic activity and standard error were calculated for each sample.

## Supplementary Materials

The following are available online at www.mdpi.com/2072-6651/9/12/389/s1, Figure S1: SDS-PAGE analyses of purified recombinant proteins. Prior to structural studies, purified recombinant proteins were analyzed by 20% SDS-PAGE: Molecular mass markers-14 kDa-45 kDa are displayed; Lanes 1–3 CaMWt; Lanes 4–6 D22A; Lane 7 CyaA-ACD, Table S1: The ratio of *θ*_222_/*θ*_208_ of CaMs determined in the presence and absence CyaA-ACD.
