# Supplementary material for: Site I Inactivation Impacts Calmodulin Calcium Binding and Activation of Bordetella pertussis Adenylate Cyclase Toxin
Source: Toxins (Basel). 2017 Nov 30;9(12):389. doi: 10.3390/toxins9120389 (PMC5744109; doi:10.3390/toxins9120389)
Supplement: Supplementary file 1 [file toxins-09-00389-s001.pdf]

Supplementary Materials: Site I Inactivation Impacts Calmodulin Calcium Binding and Activation of *Bordetella Pertussis* Adenylate Cyclase Toxin

Christian W. Johns and Natosha L. Finley

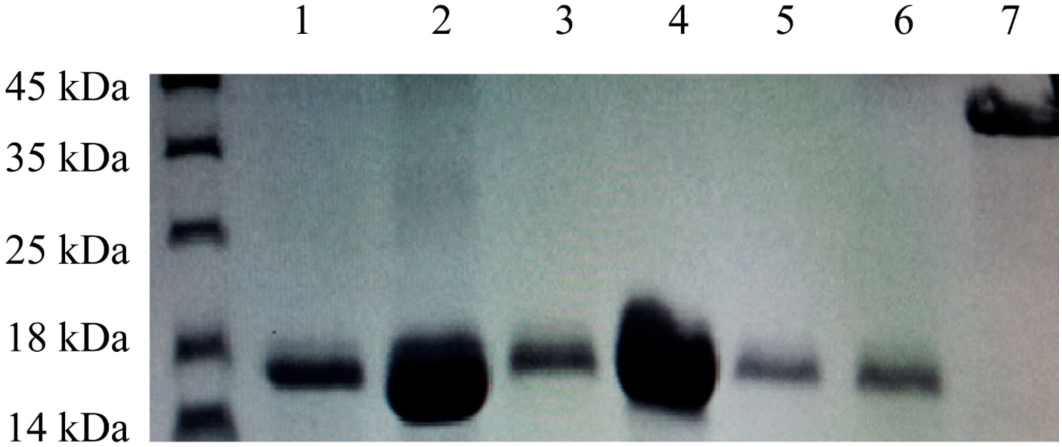

Figure S1. SDS-PAGE analysis of purified recombinant proteins. Prior to structural studies, purified recombinant proteins were analyzed by 20% SDS-PAGE: Molecular mass markers-14 kDa-45 kDa are displayed; Lanes 1–3 CaMWt; Lanes 4–6 D22A; Lane 7 CyaA-ACD.

Table S1. The ratio of $\theta_{222}/\theta_{208}$ of CaMs determined in the presence and absence CyaA-ACD.

Sample	θ_{208} (mdeg)	θ_{222} (mdeg)	$\theta_{222}/\theta_{208}$
CaMWt	−86.88	−86.00	0.99
D22A	−77.91	−88.73	1.13
CyaA-ACD	−86.93	−78.06	0.75
CaMWt/CyaA-ACD	−88.08	−83.24	0.94
D22A/CyaA-ACD	−92.95	−83.44	0.89
